# What determines subjective health status in patients with chronic obstructive pulmonary disease: importance of symptoms in subjective health status of COPD patients

**DOI:** 10.1186/1477-7525-6-115

**Published:** 2008-12-18

**Authors:** Signe Berit Bentsen, Anne Hildur Henriksen, Tore Wentzel-Larsen, Berit Rokne Hanestad, Astrid Klopstad Wahl

**Affiliations:** 1 Stord/Haugesund University College, Department of Nursing Education, Haugesund, Norway; 2 Learning and Coping Centre, Haugesund Hospital, Haugesund, Norway; 3 Department of Respiratory Medicine, University Hospital of Trondheim, Trondheim, Norway; 4 Centre for Clinical Research, Haukeland University Hospital, Bergen, Norway; 5 Department of Public Health and Primary Health Care, University of Bergen, Bergen, Norway; 6 Institute of Nursing and Health Science, University of Oslo, Oslo, Norway

## Abstract

**Background:**

Subjective health status is the result of an interaction between physiological and psychosocial factors in patients with chronic obstructive pulmonary disease (COPD). However, there is little understanding of multivariate explanations of subjective health status in COPD. The purpose of this study was to explore what determines subjective health status in COPD by evaluating the relationships between background variables such as age and sex, predicted FEV_1_%, oxygen saturation, breathlessness, anxiety and depression, exercise capacity, and physical and mental health.

**Methods:**

This study had a cross-sectional design, and included 100 COPD patients (51% men, mean age 66.1 years). Lung function was assessed by predicted FEV_1_%, oxygen saturation by transcutaneous pulse oximeter, symptoms with the St George Respiratory Questionnaire and the Hospital Anxiety and Depression Scale, physical function with the Incremental Shuttle Walking Test, and subjective health status with the SF-36 health survey. Linear regression analysis was used.

**Results:**

Older patients reported less breathlessness and women reported more anxiety (p < 0.050). Women, older patients, those with lower predicted FEV_1_%, and those with greater depression had lower physical function (p < 0.050). Patients with higher predicted FEV_1_%, those with more breathlessness, and those with more anxiety or depression reported lower subjective health status (p < 0.050). Symptoms explained the greatest variance in subjective health status (35%–51%).

**Conclusion:**

Symptoms are more important for the subjective health status of patients with COPD than demographics, physiological variables, or physical function. These findings should be considered in the treatment and care of these patients.

## Background

Chronic obstructive pulmonary disease (COPD) is a progressive lung disease characterized by impairment of lung function with airway obstruction, which is most frequently the result of tobacco smoke [1]. COPD is one of the major causes of morbidity and mortality worldwide. Many people suffer from this disease for years and die from it or its complications [1]. Hoogendoorn et al. [2] estimated that the prevalence of diagnosed COPD, the number of deaths, and the associated health costs will increase during the next decade. In addition to the social strain, COPD also influences the patients' symptoms, function, and subjective health status [3].

An important issue in understanding the complexity of COPD as an illness and thereby its management, is what determines the subjective health status of these patients. Wilson and Cleary [4] suggested a model that clarified the relationships between biological and physiological variables, symptoms, function, general health perception, and overall quality of life, and the impact of the characteristics on individuals and their environments. This model indicated that biological and physiological processes affect the perception of symptoms, which in turn affects function, general health perception, and overall quality of life. However, these authors point out that this main causal direction in their model does not imply that there are not reciprocal relationships [4].

Several studies of COPD patients have examined different associations between physiological variables, symptoms, physical function, and subjective health status. For example, de Torres et al. [5] investigated differences in physiological factors and sex, and reported that women have better oxygen saturation than men [5]. In terms of symptoms, studies of COPD patients have shown that higher oxygen consumption is associated with improved mood, and lower predicted FEV_1_% is associated with more breathlessness [6,7]. Furthermore Cleland et al [8] found that older COPD patients report less anxiety and depression than younger. Anderson [6] found that greater depression is associated with lower physical function. With regard to subjective health status, studies have reported that women suffering from COPD and older COPD patients report worse physical health [5,9,10]. Other studies have reported that lower predicted FEV_1_% and functional exercise capacity and greater anxiety and depression are associated with lower subjective health status [8,11-13].

**Figure 1:**
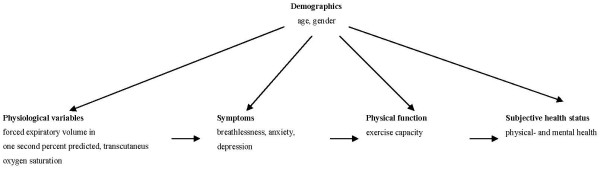
**A proposed model for the relationships between demographics, physiological variables, symptoms, physical function and subjective health status**.

The abovementioned studies mainly investigated bivariate relationships between demographics, physiological variables, symptoms, physical function, and subjective health status, but lack a multivariate perspective on subjective health status in COPD. According to the biopsychosocial perspective, subjective health status cannot be explained by biological and physiological factors alone. Instead, subjective health status is the result of an interaction between physiological and psychosocial factors [14]. COPD is a chronic disease, which must be managed rather than cured. Therefore, knowledge about what determines subjective health status in this group of patients is relevant for the treatment of COPD, and for the care and rehabilitation of patients. To this end, the aim of the present study was to explore the determinants of subjective health status in COPD by evaluating the relationships between background variables such as age and sex, predicted FEV_1_%, oxygen saturation, breathlessness, anxiety and depression, exercise capacity, and physical and mental health. Based on previous studies in COPD patients and the conceptual model of Wilson and Cleary, the following conceptual model is postulated (Figure 1).

## Methods

### Design, sample, and data collection

This study had a cross-sectional design, and included 136 patients with COPD recruited from the outpatient clinic at a medium-sized hospital between August 2005 and August 2007. The patients were referred to the out-patient clinic to attend a rehabilitation programme designed for COPD patients. Those who fulfilled the criteria listed below were invited to participate in this study.

Inclusion criteria for the study

• Age > 35 years

• Diagnosed with COPD by a respiratory physician

• Symptoms such as breathlessness, chronic cough, and sputum production

• FEV1/FVC < 70% and FEV1 < 80% predicted

• Able to read and write Norwegian

Exclusion criteria for the study

• Using long-term oxygen treatment

• Unstable heart disease

Patients were given verbal and written information about the study, an informed consent form giving their permission to take part in the study, and a questionnaire with a hand-signed cover letter and a pre-stamped envelope when they underwent the examination at the out-patient clinic. Each patient's respiratory symptoms and physical health were assessed by a physician, nurse, and physiotherapist, all specialized in pulmonary disease. All patients underwent height and weight measurements, spirometry, an Incremental Shuttle Walking Test (ISWT), and electrocardiogram. Those who had not returned the questionnaire within two weeks were sent a reminder. This study was performed according to the Declaration of Helsinki and was approved by the hospital unit, the Regional Committee for Medical Research Ethics, and the Norwegian Social Science Data Services.

### Measures

The measurements described below were used to examine demographics, physiological variables, symptoms, physical function, and subjective health status.

#### (A) Demographics

The patients completed a questionnaire consisting of the following variables: age (continuous variable, in years) and sex.

#### (B) Physiological variables

Data on lung function and transcutaneous oxygen saturation were collected during the visit at the out-patient clinic.

##### Pulmonary function tests

Spirometry was performed with a Vitalograph Alpha spirometer, according to international guidelines [15]. Forced expiratory volume in one second (FEV_1_) and forced vital capacity (FVC) were measured and the predicted values calculated according to a Norwegian reference population [16]. FEV1/FVC% was calculated and a value < 0.7 together with FEV_1 _< 80% predicted was used as a diagnostic criterion for COPD. FEV_1_(litre) and FEV_1 _as a percentage of the predicted value (predicted FEV_1_%) were used as a measure of lung function.

##### Oxygen saturation

Transcutaneous oxygen saturation (SaO_2_%) was measured with a Konica Minolta PulsOx-3i Pulse Oximeter. SaO_2_% was measured immediately before the incremental shuttle walking test [17].

#### (C) Symptoms

To measure their symptoms, the patients filled out a questionnaire on breathlessness, anxiety, and depression.

##### Breathlessness

Breathlessness was measured with the St George's Respiratory Questionnaire (SGRQ) [18]. The SGRQ is a disease-specific instrument for patients suffering from pulmonary disease. The questionnaire consists of 76 items divided into three components: 1) symptoms, 2) activity, and 3) impact. A sum is calculated for each component. Each of the scores ranges from 0 to 100, the lower scores indicating better health status [19-21]. The SGRQ has been translated into different languages and used in several studies of COPD patients, including in Norway [22,23]. The questionnaire has been tested for reliability and validity in different studies and the results showed satisfactory reliability and validity in COPD patients [24-26]. Only the symptom component, which measures breathlessness in terms of frequency and distress [18], was used in this study. The symptom component consists of 8 items including frequencies and distress of breathlessness in term of phlegm/sputum, shortness of breath, wheezing and chest trouble [18,21].

##### Anxiety and depression

Anxiety and depression were measured with the Hospital Anxiety and Depression Scale (HADS). HADS is a questionnaire developed to measure anxiety and depression in non-psychiatric patients treated at hospital clinics. The questionnaire consists of 14 items. Seven items measure anxiety (HADS-A) and seven items measure depression (HADS-D). The items are scored on a four-step scale ranging from 0 (not at all) to 3 (very much). One anxiety and one depression scale are scored by summing the patient's responses. The scores range from 0–21, with higher scores indicating higher anxiety and depression [27,28]. HADS has been thoroughly tested for psychometric properties [27-30] and has been used in patients suffering from COPD and the general population in Norway [31-33].

#### (D) Physical function

Data on physical function were collected during the examination at the out-patient clinic.

##### Exercise capacity

Exercise capacity was measured with the ISWT. The ISWT is a standardized progressive walking test used to measure functional exercise capacity in patients with cardiorespiratory conditions. The test requires patients to walk at increasing speeds up and down a 10-metre course. The speed of walking increases every minute and is controlled by audio signals played on a DVD. The distance walked is reported in metres and greater distances indicate better exercise capacity [34]. The ISWT has shown satisfactory reliability and validity in COPD patients [34,35].

#### (E) Subjective health status

##### Physical and mental health

The Short Form 36 health survey (SF-36) was used to measure physical and mental health. SF-36 is one of several generic questionnaires developed in the United States by the Medical Outcomes Study to assess subjective health status [36]. The questionnaire consists of 36 questions that measure eight conceptual components: physical functioning, physical role limitations, bodily pain, self-reported general health, vitality, social function, emotional role limitations, and mental health. The scores in each component and the total scores are transformed onto 0–100 scales. Higher scores indicate better subjective health status [36]. One physical health summary score and one mental health summary score were computed from the eight dimension scores. The physical health summary score is mainly based on the physical health, physical role limitations, bodily pain, and general health components, whereas the mental health summary score is mainly based on the vitality, social function, emotional role limitations, and mental health components [37]. In this study, we used the physical and mental health summary scores. The questionnaire has shown satisfactory reliability and validity in COPD patients, and has been thoroughly tested for psychometric properties in several countries, including Norway [38-41].

### Statistical analysis

The data were analysed with SPSS for Windows version 15.0 (SPSS Inc., Chicago, IL, USA). Missing data for the SF-36 and SGRQ were accommodated according to the user manuals [21,36]. For the HADS, missing data were accommodated for individuals who had responded to five or more of the seven items of HADS-A or HADS-D [30]. Descriptive analyses (mean, standard deviation [SD], range) were used. Simple and multiple linear regression analyses were used to investigate the relationships between demographics, physiological variables, symptoms, physical function, and subjective health status. In the multiple linear regressions, the analysis demographics were entered as independent variables. Physiological variables, symptoms, and physical function values were used as both independent and dependent variables, and subjective health status was entered as a dependent variable according to the model shown in Figure 1. In the present study, p < 0.05 was considered statistically significant.

## Results

### Descriptive

The sample consisted of 100 (response rate, 74%) patients suffering from COPD and awaiting participation in an outpatient pulmonary rehabilitation programme. The characteristics of the responders are shown in Table 1.

**Table 1:** Characteristics of the responders (N = 100)

	N	(%)	Mean	(SD)	Range
Age (years)			66.1	(8.3)	42–82
Gender					
Male	51	(51)			
Female	49	(49)			
Spirometry					
FEV_1 _(litre)^a^			1.31	(0.50)	0.42–2.54
FEV_1_% predicted^a^			46.0	(15.0)	16–79
FEV_1_/FVC%^a^			51.6	(12.5)	28–69
Transcutaneus oxygen saturation (SaO_2_%)^a^			96.0	(1.9)	88–99
Breathlessness (SGRQ)^b^					
(0–100)			49.8	(27.8)	0.0–97.5
Anxiety (HADS-A)^b^					
(0–21)			5.9	(3.9)	0.0–17.0
Depression (HADS-D)^b^					
(0–21)			4.5	(3.7)	0.0–19.0
Exercise capacity (ISWT)^a^					
(metre)			336.7	(163.9)	57.0–770.0
Physical health summary scale (SF-36)^a^			38.4	(9.9)	14.7–58.2
Mental health summary scale(SF-36)^a^			48.6	(10.4)	20.8–68.3

^a^Higher score indicate better lung function, oxygen saturation, exercise capacity and physical and mental health.^b^Higher score indicate more breathlessness, anxiety and depression.

### Relationships between age, sex, physiological variables, and symptoms

In the bivariate analysis, age (regression coefficient = -0.75, p = 0.025) and predicted FEV_1_% (regression coefficient = -0.42, p = 0.024) showed a significant relationship to breathlessness, and sex (difference = -1.86, p = 0.017) to anxiety (level 0, Additional file 1). When both demographic and physiological variables were entered in the analysis, age (regression coefficient = -0.84, p = 0.019) and sex (difference = -2.21, p = 0.011) still showed a significant relationship to breathlessness and anxiety (level 2, Additional file 1).

### Relationships between age, sex, physiological variables, symptoms, and physical function

Age (regression coefficient = -7.12, p = 0.001), predicted FEV_1_% (regression coefficient = 2.97, p = 0.015), anxiety (regression coefficient = -9.22, p = 0.041), and depression (regression coefficient = -16.26, p < 0.001) showed significant bivariate relationships to exercise capacity (level 0, Additional file 1). When all the variables were entered into the regression analysis, age (regression coefficient = -7.45, p < 0.001), sex (difference = 76.41, p = 0.022), predicted FEV_1_% (regression coefficient = 2.71, p = 0.020), and depression (regression coefficient = -14.22, p = 0.009) showed significant relationships to exercise capacity (level 3, Additional file 1).

### Relationships between age, sex, physiological variables, symptoms, physical function, and subjective health status

In the bivariate analysis, predicted FEV_1_% (regression coefficient = 0.19, p = 0.007), breathlessness (regression coefficient = -0.17, p < 0.001), anxiety (regression coefficient = -1.04, p < 0.001), depression (regression coefficient = -1.54, p < 0.001), and exercise capacity (regression coefficient = 0.02, p = 0.021) were significantly associated with physical health (level 0, Additional file 1). When demographics, physiological variables, symptoms, and physical function were entered into the analysis, only breathlessness (regression coefficient = -0.09, p = 0.027) and depression (regression coefficient = -0.88, p = 0.015) were significantly associated with physical health (level 4, Additional file 1).

Our results also showed significant bivariate relationships between anxiety (regression coefficient = -1.74, p < 0.001), depression (regression coefficient = -1.80, p < 0.001), exercise capacity (regression coefficient = 0.02, p = 0.031), and mental health (level 0, Additional file 1). When all the variables were entered into the regression analysis, predicted FEV_1_% (regression coefficient = -0.14, p = 0.043), anxiety (regression coefficient = -0.85, p = 0.004), and depression (regression coefficient = -1.31, p < 0.001) showed significant relationships to mental health (level 4, Additional file 1).

Age and sex account for only -1% and 1%, respectively, of the adjusted R^2 ^for physical and mental health. When the physiological variables were entered into the model, the adjusted R^2 ^increased to 1% for physical health and 2% for mental health. When symptoms were added, the explained variance increased to 36% for physical health and 53% for mental health, whereas physical function added no substantial variance. When all the variables were entered into the regression analysis, the explained variance was 37% for the physical health component and 53% for the mental health component (levels 1–4, Additional file 1).

### Internal consistence

In this study, Cronbach's alpha was 0.86, 0.85, and 0.87 for the symptom, activity, and impact components, respectively, and 0.93 for the total score of the SGRQ. With regard to HADS, Cronbach's alpha was 0.85 for anxiety and 0.84 for depression. Cronbach's alpha ranged from 0.77 to 0.90 for SF-36 subscales. The lowest value was observed for the general health component (0.77) and the highest value for the bodily pain component (0.90).

## Discussion

The results of this study show that patients with more breathlessness and depression reported lower physical health. Moreover, those with better lung function but more anxiety and depression reported lower mental health. These results also show that symptoms explain a greater proportion of the variance in subjective health status than do demographics, physiological variables, or physical function. According to the biopsychosocial model, no one single factor explains the subjective health status. Instead, it reflects the complexity of the associations between biological and psychosocial factors, progresses of symptoms, to clusters of symptoms, to syndromes, and finally to diseases with specific pathogeneses and pathology [14].

This is the first study to explore a multivariate perspective on subjective health status in COPD patients based on Wilson and Cleary's [4] conceptual model of biopsychosocial relationships to subjective health status. In this study, a conceptual model was established based on Wilson and Cleary's framework and previous COPD-specific studies. In the model, there is a unidirectional relationship between the biological and physiological variables, symptoms, and physical function, which leads to the subjective health status (Figure 1). According to Osoba [42], there is a reasonably strong correlation between the proximal components of Wilson and Cleary's model (such as symptoms and physical function) and a weaker correlation between the more distant components (such as the physiological variables and subjective health status). There may also be a bidirectional relationship between some components [42]. There is not necessarily a strong association between the objective physiological indicators of the disease and the patients' subjective experience of their health status. In this respect, studies of COPD patients have found weak associations between objective measures of disease, symptoms, physical function, and subjective health status [11,13,22,43].

### Relationships between age, sex, and physiological variables

The results of this study show insignificant associations between age, sex, and oxygen saturation. Conflicting results have been found in previous studies. De Torres et al. [5] found that women suffering from COPD tended to have better oxygen saturation than men. Conversely, Di Marco et al. [43] found an insignificant association between sex and oxygen saturation. Insignificant associations between age, sex, and oxygen saturation suggest that the women and men studied were at the same stage of COPD [5,44].

### Relationships between age, sex, physiological variables, and symptoms

The observation that older COPD patients report less breathlessness than younger is in contrast to Stavem et al [45] who not find any such association. This finding may be due to response shift [46]. Patients adapt over time in relation to goals, expectations and values, and their perceptions of symptoms may therefore change. Furthermore, the process of learning to cope with health problems is well-known in chronically ill patients [46]. Older COPD patients may have suffered longer from COPD and anticipate illness as part of growing old. Moreover, health- related stressors may not produce the same reactions in elderly. Although older patients may have difficulties due to breathlessness, they may see physical and functional disability as result in growing older [8,47]. The fact that women tend to report more anxiety than men is not surprising because there is ample evidence of a higher prevalence of anxiety among woman than among men [48,49]. That women report more anxiety than men is also consistent with previous studies of COPD patients [13,43]. In this study, small and insignificant associations were identified between physiological variables and symptoms. These results are in accordance with previous studies of COPD patients, which found small and insignificant associations between physiological measurements and breathlessness, anxiety, and depression[7,11,22,43,45].

### Relationships between age, sex, physiological variables, symptoms, physical function, and subjective health status

Patients with less breathlessness and depression reported better physical health, and those with less anxiety and depression reported better mental health, which is consistent with previous studies of COPD patients [8,45,50]. However, it is surprising that lung function was not associated with physical health and that better lung function was associated with worse mental health. The same trend was observed in other studies of COPD patients, although the association was not statistically significant [45,51]. The results of our study show that the association between symptoms and subjective health status was stronger than the association between physiological variables and subjective health status, and this supports the multidimensional impact of COPD on subjective health status [42]. Furthermore, the fact that subjective health status represents something other than physiological and pathological factors is useful information for consideration in the treatment and care of COPD patients [7,45,52].

### Limitations

In this study, age, sex, lung function, oxygen saturation, breathlessness, anxiety, depression, and exercise capacity influenced subjective health status. However, according to previous studies of COPD patients, body mass index, education, social status, sleeping habits, and co-morbidity could be important supplementary factors affecting subjective health status in this sample [10,12,13]. This study is limited to some degree. The sample size was quite small, which restricts the number of factors included in the multivariate testing of subjective health status [53]. Because of the cross-sectional design, no absolute conclusions can be drawn about causality or the directions of the relationships between many of the variables [54]. The patients included in this study were awaiting participation in a pulmonary rehabilitation programme, and were thus not a representative sample of all COPD patients. The strength of this study is its multivariate approach to explaining subjective health status. According to the biopsychosocial model, subjective health status is associated with physiological factors as well as symptoms and psychosocial factors [14].

### Implications for clinical practice

The results of this study indicate that symptoms are very important to patients' subjective health status, which in turn supports the view that a pulmonary rehabilitation programme focusing on the management of symptoms, such as breathlessness, anxiety, and depression, is required to alleviate symptoms and increase subjective health status[55].

A model that explains the relationships between different outcomes is important in clinical practice to correctly interpret the results of outcome assessments [4,42]. For example, if subjective health status is determined by symptoms and physical function, then symptoms and physical function should be treated [42]. In COPD, symptoms such as breathlessness, anxiety, and depression are usually evident before there is a reduction in subjective health status. However, it is more difficult to determine the causal direction between breathlessness, anxiety, depression, and physical function, and as breathlessness, anxiety, and depression may be caused by a decrease in function [52,56].

## Conclusion

When controlled for all variables, more breathlessness and depression were associated with lower physical health, and better lung function, and greater anxiety and depression were associated with a lower mental health, with symptoms explaining the greatest variance. These findings highlight the importance of rehabilitation programmes that focus on the management of symptoms in relation to COPD.

## Competing interests

The authors declare that they have no competing interests.

## Authors' contributions

SBB conceived and design the study, collected the date, performed statistical analysis and drafted the manuscript. AKW, BRH and AHH participated in the design and revised the manuscript critically. TWL participated in the design, conducted the statistical analyses and revised the manuscript critically. All authors read and approved the final manuscript.

## Supplementary Material

Additional File 1 **Table 2.** The relationships between independent and dependent variables by linear regression analyses (Level 0–4 : regressionscoefficients; Level 0: bivariate analysis, Level 1–4: multivariate analysis). Click here for file
