# Supplementary material for: What determines subjective health status in patients with chronic obstructive pulmonary disease: importance of symptoms in subjective health status of COPD patients
Source: Health Qual Life Outcomes. 2008 Dec 18;6:115. doi: 10.1186/1477-7525-6-115 (PMC2640371; doi:10.1186/1477-7525-6-115)
Supplement: Additional File 1 — Table 2. The relationships between independent and dependent variables by linear regression analyses (Level 0–4 : regressionscoefficients; Level 0: bivariate analysis, Level 1–4: multivariate analysis). [file 1477-7525-6-115-S1.doc]

Table 2: The relationships between independent and dependent variables by linear regression analyses (Level 0-4 : regressionscoefficients; Level 0: bivariate analysis, Level 1-4: multivariate analysis)

|  | **Physiological variables** | | | | **Symptoms** | | | | | | | | |
| --- | --- | --- | --- | --- | --- | --- | --- | --- | --- | --- | --- | --- | --- |
|  | **FEV_1_ % pred (0-100)^b^** | | **SaO_2_ % (0-100)^b^** | | **SGRQ (0-100)^c^** | | | **HADS-A (0-21)^c^** | | | **HADS-D (0-21)^c^** | | |
|  | **Level 0** | **Level 1** | **Level 0** | **Level 1** | **Level 0** | **Level 1** | **Level 2** | **Level 0** | **Level 1** | **Level 2** | **Level 0** | **Level 1** | **Level 2** |
| **Demographics** |  |  |  |  |  |  |  |  |  |  |  |  |  |
| Age (years) | 0.27 | 0.32 | -0.01 | -0.01 | **-0.75**  ***** | **-0.84**  ***** | **-0.84**  ***** | -0.05 | -0.03 | -0.02 | 0.01 | 0.01 | 0.04 |
| Gender (men)^a^ | -3.35 | -4.35 | -0.55 | -0.54 | 5.59 | 8.19 | 6.18 | **-1.86**  ***** | **-1.77**  ***** | **-2.21**  ***** | -0.04 | -0.07 | -0.32 |
| **Physiological variables** |  |  |  |  |  |  |  |  |  |  |  |  |  |
| FEV_1_% pred (0-100)^b^ |  |  |  |  | **-0.42**  ***** |  | -0.28 | -0.01 |  | -0.02 | -0.04 |  | -0.05 |
| SaO_2_% (0-100)^b^ |  |  |  |  | -1.63 |  | -0.75 | -0.13 |  | -0.16 | -0.10 |  | 0.03 |
| **Symptoms** |  |  |  |  |  |  |  |  |  |  |  |  |  |
| SGRQ (0-100)^c^ |  |  |  |  |  |  |  |  |  |  |  |  |  |
| HADS-A (0-21)^c^ |  |  |  |  |  |  |  |  |  |  |  |  |  |
| HADS-D (0-21)^c^ |  |  |  |  |  |  |  |  |  |  |  |  |  |
| **Physical function** |  |  |  |  |  |  |  |  |  |  |  |  |  |
| ISWT (metre)^b^ |  |  |  |  |  |  |  |  |  |  |  |  |  |
| **Adjusted R^2^** |  | **0.02** |  | **0.00** |  | **0.05** | **0.06** |  | **0.04** | **0.04** |  | **-0.02** | **-0.01** |

P-value=*P<0.05, **P<0.01, ***P<0.001. FEV_1_ % pred=forced expiratory volume in one second percent of predicted value, SaO_2_%=transcutaneus oxygen saturation, SGRQ=breathlessness, HADS-A=anxiety, HADS-D=depression, ISWT=exercise capacity, SF-36-PCS=physical health, SF-36-MCS=mental health. ^a^Women as reference category. ^b^Higher score indicate better lung function, oxygen saturation, exercise capacity, and physical and mental health. ^c^Higher score indicate more breathlessness, anxiety and depression.

Table 2 continued

|  | **Physical function** | | | | **Subjective health status** | | | | | | | | | |
| --- | --- | --- | --- | --- | --- | --- | --- | --- | --- | --- | --- | --- | --- | --- |
|  | **ISWT (metre)^b^** | | | | **SF-36 – PCS^b^** | | | | | **SF-36 – MCS^b^** | | | | |
|  | **Level 0** | **Level 1** | **Level 2** | **Level 3** | **Level 0** | **Level 1** | **Level 2** | **Level 3** | **Level 4** | **Level 0** | **Level 1** | **Level 2** | **Level 3** | **Level 4** |
| **Demographics** |  |  |  |  |  |  |  |  |  |  |  |  |  |  |
| Age (years) | **- 7.12**  ****** | **- 7.64**  ******* | **- 8.18**  ******* | **-7 .45**  ******* | 0.13 | 0.14 | 0.10 | -0.04 | 0.05 | -0.05 | -0.09 | -0.12 | -0.21 | -0.17 |
| Gender (men)^a^ | 43.23 | 61.18 | **77.46**  ***** | **76.41**  ***** | -0.08 | -0.52 | -0.30 | -0.86 | - 1.73 | 3.59 | 3.88 | 4.76 | 2.97 | 2.54 |
| **Physiological variables** |  |  |  |  |  |  |  |  |  |  |  |  |  |  |
| FEV_1_% pred (0-100)^b^ | **2.97**  ***** |  | **3.32**  ****** | **2.71**  ***** | **0.19**  ****** |  | 0.10 | 0.06 | 0.03 | - 0.10 |  | -0.10 | -0.12 | **-0.14**  ***** |
| SaO_2_% (0-100)^b^ | 15.73 |  | 7.43 | 8.23 | 0.95 |  | 0.64 | 0.36 | 0.25 | - 0.09 |  | 0.43 | -0.04 | -0.10 |
| **Symptoms** |  |  |  |  |  |  |  |  |  |  |  |  |  |  |
| SGRQ (0-100)^c^ | 0.00 |  |  | 0.14 | **-0.17**  ******* |  |  | **-0.09**  ***** | **- 0.09**  ***** | -0.06 |  |  | -0.05 | -0.05 |
| HADS-A (0-21)^c^ | **- 9.22**  ***** |  |  | 1.13 | **-1.04**  ******* |  |  | -0.47 | - 0.50 | **-1.74**  ******* |  |  | **-0.83**  ****** | **-0.85**  ****** |
| HADS-D (0-21)^c^ | **-16.26**  ****** |  |  | **-14.22**  ****** | **-1.54**  ******* |  |  | **-1.03**  ****** | **- 0.88**  ***** | **-1.80**  ******* |  |  | **-1.39**  ******* | **-1.31**  ******* |
| **Physical function** |  |  |  |  |  |  |  |  |  |  |  |  |  |  |
| ISWT (metre)^b^ |  |  |  |  | **0.02**  ***** |  |  |  | 0.01 | **0.02**  ***** |  |  |  | 0.01 |
| **Adjusted R^2^** |  | **0.15** | **0.24** | **0.31** |  | **-0.01** | **0.01** | **0.36** | **0.37** |  | **0.01** | **0.02** | **0.53** | **0.53** |
